# Mechanism of diastereoisomer-induced chirality of BiOBr†

**DOI:** 10.1039/d1sc05601h

**Published:** 2022-02-04

**Authors:** Kun Ding, Jing Ai, Yingying Duan, Lu Han, Zhibei Qu, Shunai Che

**Affiliations:** School of Chemistry and Chemical Engineering, Frontiers Science Center for Transformative Molecules, State Key Laboratory of Composites Materials, Shanghai Key Laboratory for Molecular Engineering of Chiral Drugs, Shanghai Jiao Tong University 800 Dongchuan Road Shanghai 200240 P. R. China chesa@sjtu.edu.cn; School of Chemical Science and Engineering, Tongji University 1239 Siping Road Shanghai 200092 P. R. China yyduan@tongji.edu.cn luhan@tongji.edu.cn; Department of Medicinal Chemistry, School of Pharmacy, Fudan University 826 Zhangheng Road Shanghai 201203 P. R. China quzhibei@gmail.com

## Abstract

Chiral molecule-driven asymmetric structures are known to be elusive because of the intriguing chirality transfer from chiral molecules to achiral species. Here, we found that the chiral assembly of BiOBr is independent of the chirality of the organic molecular inducer but dependent on geometric structural matching between the inducer and inorganic species. Diastereoisomeric sugar alcohols (DSAs) with identical numbers of carbon chiral centers and functional groups but with different *R*/*S* configurations and optical activities (OAs) were chosen as symmetry-breaking agents for inducing chiral mesostructured BiOBr films (CMBFs) under hydrothermal conditions. Multiple levels of chirality with different handedness were identified in the CMBFs. Density functional theory (DFT) calculations and molecular dynamics (MD) simulations suggest that asymmetric defects in the Br–Bi tetragonal cone caused by physically adsorbed DSAs on the surfaces of the BiOBr crystals are the geometric basis for triggering the chiral twist in the BiOBr monolayer. Our findings provide new insights for understanding the origin of chirality and the chiral transfer mechanism underlying the assembly of achiral species.

## Abstract 2

The chirality transfer is dependent on geometrical matching between the chiral inducer and inorganic species.

## Graphical abstract

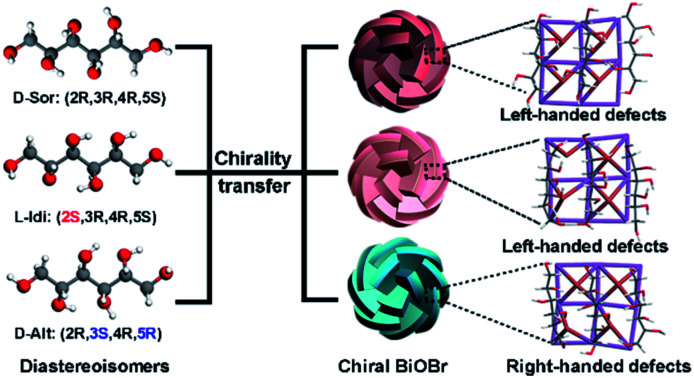

## Introduction

Chiral molecule-driven asymmetric structures are ubiquitous in biological organisms^1–3^ and have been artificially mimicked to fabricate chiral materials, including organics,^4–9^ inorganics,^10–16^ and organic–inorganic hybrids.^17–19^ The great potential of assembled chiral inorganics in optics, electricity, magnetism, biology, and chemistry has attracted tremendous interest in the chiral molecule-driven assembly of chiral inorganic nanostructures.^20–24^

Although the chemical and physical chirality origins of inorganic nanostructures involving chiral crystal surfaces,^25–28^ screw dislocation,^29–31^ chiral imprinting,^32,33^ and chiral geometries and assemblies induced by chemisorbed chiral molecules have been proposed,^13–15,34,35^ chirality transfer from the molecular level to the macroscopic level of inorganic nanostructures across nanometers, micrometers and beyond is still not well understood.^36^ Diastereoisomers with the same number of carbon chiral centers and functional groups but different *R*/*S* configurations and OAs would be the most noteworthy candidate molecules for studying the intriguing relationship between chiral molecular inducers and inorganic species.

## Results and discussion

### Chirality of diastereoisomer inducers

**Fig. 1:**
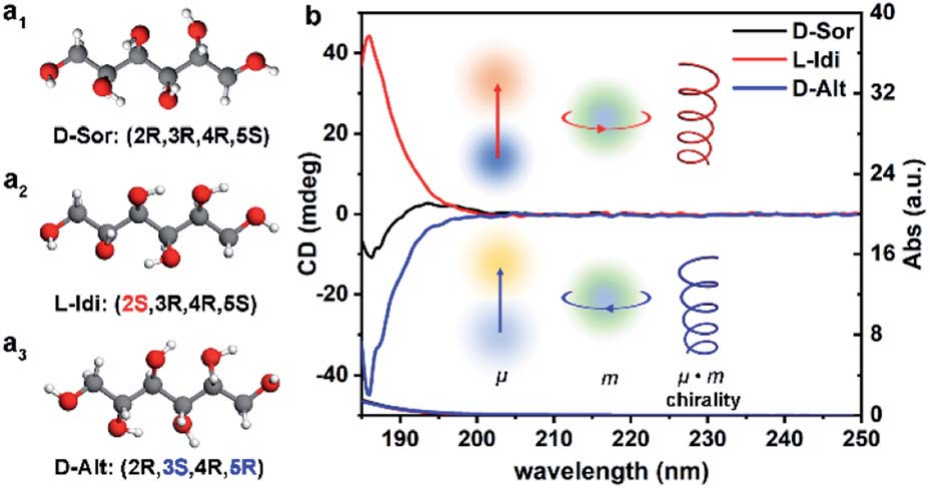
Optimized structural models (a) and transmitted UV/Vis (TUV/Vis) and TCD spectra (b) of three types of DSA solutions with a concentration of 5.5 × 10^−3^ M.

Fig. 1a shows the optimized structural models of DSAs with the same chemical formula (C_6_H_14_O_6_) and different configurations, d-sorbitol (d-Sor), l-iditol (l-Idi) and d-altritol (d-Alt), with configurations of (2*R*,3*R*,4*R*,5*S*), (2*S*,3*R*,4*R*,5*S*) and (2*R*,3*S*,4*R*,5*R*), respectively. As expected from their names, d-Sor and d-Alt exhibit negative transmitted circular dichroism (TCD) signals, while l-Idi exhibits a positive TCD signal at wavelengths of 185–200 nm (Fig. 1b). The ordinate on the CD spectra is defined as the ellipticity (*θ* ∝ *ε*_L_ − *ε*_R_) at the particular absorption bands, which is determined by a combination vector of the electric dipole transition moment (*μ*) and magnetic dipole transition moment (*m*) induced by incident circularly polarized electromagnetic waves.^37,38^ Thus, the spectra can show deviations from the baseline that are positive, negative or zero, indicating their different chiralities.

### Chirality of CMBFs

**Fig. 2:**
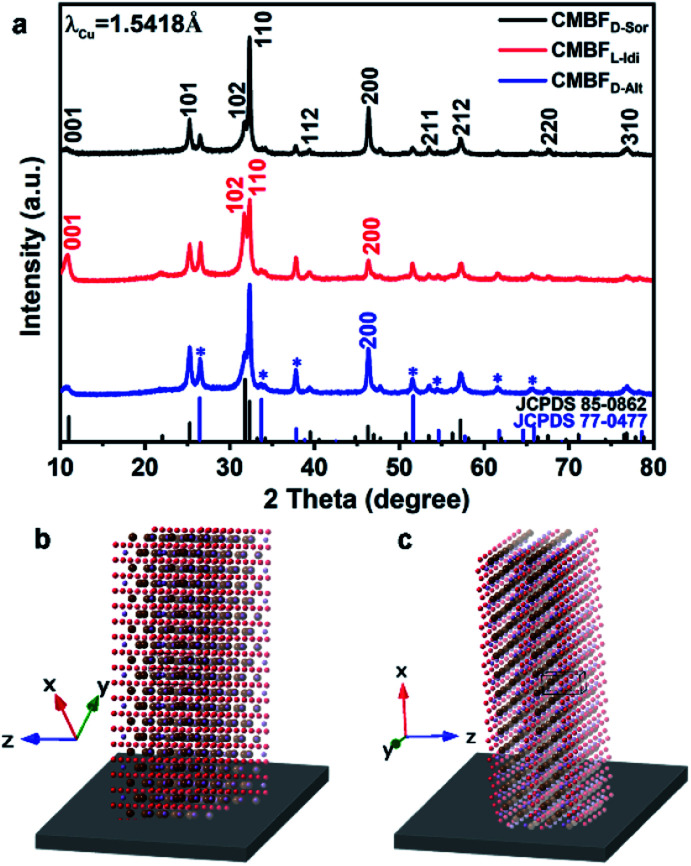
XRD patterns of CMBFs synthesized using different DSAs (a). The growing direction of BiOBr nanoflakes along the FTO substrate in CMBF_d-Sor_ and CMBF_d-Alt_ (b), and CMBF_l-Idi_ (c). The reflections marked with asterisks are attributed to the SnO_2_ (JCPDS No. 77-0477) layers on the substrate.

Here, CMBFs with different chiralities were fabricated through a hydrothermal method from a homogeneous solution composed of DSA, bismuth and bromide salts.^39^ DSAs (Fig. S1†) were selected as symmetry-breaking agents owing to the presence of multiple structurally variable optical isomers and abundant hydroxyl interaction sites with BiOBr.^40^ The bismuth and bromide ions combine in solution to generate BiOBr nanoparticles on the activated fluorine-doped tin oxide (FTO) surface and then gradually grow into dense films as the reaction proceeded (Fig. S2a–d†). The DSAs in the CMBFs were completely removed by several washes. Fourier transform infrared (FTIR) spectroscopy, Raman spectroscopy, solid-state ^13^C CP/MAS NMR spectroscopy (Fig. S4 and S5†), and thermogravimetric analysis (TG) curves (Fig. S6†) indicate that the surfaces of the CMBFs are free of chiral ligands. The CMBFs synthesized with d-Sor, l-Idi, and d-Alt were denoted as CMBF_d-Sor_, CMBF_l-Idi_, and CMBF_d-Alt_, respectively. The wide-angle X-ray diffraction (XRD) reflections of all CMBFs can be well indexed to the pure tetragonal phase of BiOBr (JCPDS No. 85-0862) (Fig. 2a). The growing direction of BiOBr nanoflakes in CMBFs on FTO substrates are shown in Fig. 2b and c (*vide post*).

**Fig. 3:**
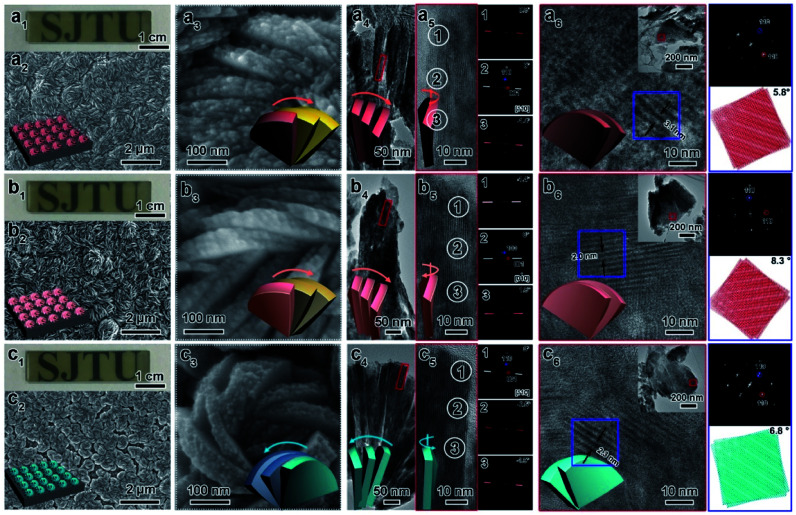
Photograph, SEM images, TEM images, FDs, and corresponding models of CMBF_d-Sor_ (a), CMBF_l-Idi_ (b), and CMBF_d-Alt_ (c) with different views. The synthetic molar composition was 0.5 DSA: 0.5 Bi(NO_3_)_3_·5H_2_O: 0.5 NaBr: 287 EG: 222 H_2_O: 7.5 × 10^−4^ NaOH.

As shown in Fig. 3a_1_–c_1_, all CMBFs have a smooth and flat surface with a translucent light-yellow color. Scanning electron microscopy (SEM) images show that the CMBFs are made up of numerous flower-shaped nanoparticles (Fig. 3a_2_–c_2_) made of bent nanoplates with lengths of 100–550 nm and widths of approximately 30–100 nm in a clockwise or anticlockwise arrangement (Fig. 3a_3_–c_3_), which is regarded as a right- or left-handed morphology (insets in Fig. 3a_3_–c_3_). This arrangement of the nanoplates is regarded as the third level of chirality of CMBFs. Moreover, the nanoplates are formed by the helical stacking of several nanoflakes with thicknesses of 8–25 nm (Fig. 3a_4_–c_4_), which is realized as the second level of chirality (see also the description of Fig. 3a_6_–c_6_, *vide post*).

Fig. 3a_5_ and c_5_ show the transmission electron microscopy (TEM) images captured from the lateral orientation of a nanoflake, and the corresponding Fourier diffractograms (FDs) reveal that the zone axes of the nanoflakes for CMBFD-Sor and CMBFD-Alt are [110]. Therefore, the growth axis of the nanoflakes perpendicular to the substrate is along the 〈110〉 direction, while the [001] axis is parallel to the substrate (Fig. 2b). This is consistent with the XRD profile that the 110 reflection shows the strongest intensity (Fig. 2a). For CMBFL-Idi, the [0–10] axis was confirmed from the TEM image taken from the side direction and the perpendicular axis is 〈100〉 (Fig. 3b_5_). The growth of the nanoflake is along the ab plane. However, the 102 and 001 reflection intensities are increased in the XRD pattern, which could be explained by the oblique growth of the nanoflakes on the substrate (Fig. 2c). Notably, the FDs taken from three consecutive positions from the top to the bottom part in the high-resolution TEM (HRTEM) image of the nanoflake (Fig. 3a_5_–c_5_) show obvious intensity changes and successive angular changes. This phenomenon occurs upon gradual misalignment of the crystal lattice due to the continuous twisted structure, which can be attributed to the primary chirality.

Fig. 3a_6_–c_6_ show the HRTEM images of CMBFs taken from the front side ([001] direction) of the stacked nanoflakes, from which the Moiré patterns are observed, demonstrating the existence of superimposed contrast formed by the rotation of two or more layers of crystals at small angles. The satellite reflections in the FDs also suggest the double diffraction phenomenon. It is worth noting that 2D Moiré patterns can be normally observed for the overlapping of 3D crystal structures, however, the parallel fringes shown in Fig. 3a_6_–c_6_ were observed, which can be attributed to the existence of the twisted crystal structures. Therefore, structural models of the two layers of crystal twisting along the [110] axis with 5.8°, 8.3° and 6.8° rotational relationships were constructed, which are in accordance with both the periodicity and the direction of the Moiré patterns in the experimental HRTEM images (Fig. 3a_6_–c_6_). This result demonstrates that the secondary chiral alignment of the nanoflakes is performed in a fan-like format. CMBF_d-Sor_ and CMBF_l-Idi_ exhibit right-handed tertiary vortices (Fig. 3a_2_ and b_2_), which are opposite to CMBF_d-Alt_ (Fig. 3c_2_). However, it is difficult to determine the left or right-handed alignment of the secondary and primary chirality from SEM and HRTEM images, which can be further confirmed by OA analysis and theoretical calculations (*vide post*).

From the morphological and structural evolution of CMBF_d-Sor_ (Fig. S2†), it can be observed that the nanoparticles with a tertiary chiral structure have been formed at the initial stage of the reaction, which could be induced by the chiral arrangements of DSA molecules under hydrothermal conditions. With the progress of crystallization, the tertiary nanosheets were split into secondary nanoplates with the simultaneous formation of primary nanoflakes due to the continuous distortion of the lattice preventing them from remaining as a complete single crystal over a long distance (Fig. S2e†). Additionally, the synthesized CMBFs exhibited good reproducibility (Fig. S3†).

**Fig. 4:**
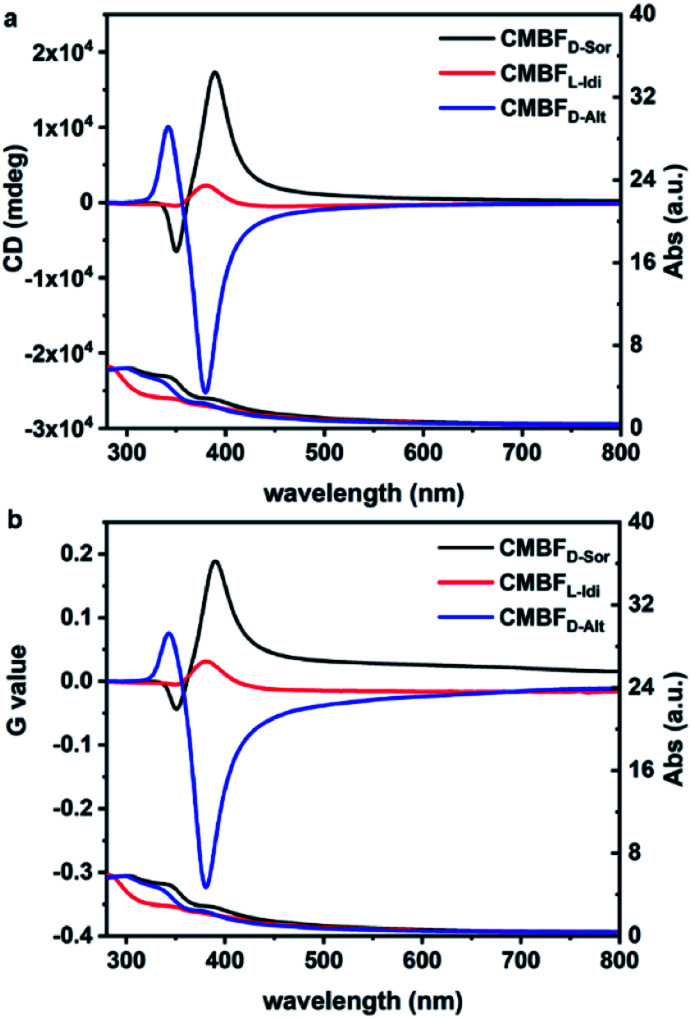
TUV/Vis and TCD spectra (a) and the corresponding Kuhn anisotropy *g*-factor values (b) of CMBFs synthesized with DSAs.

Fig. 4 shows the TUV/Vis and TCD spectra of CMBF_d-Sor_ and CMBF_l-Idi_, which exhibit strong positive signals at 360–800 nm and weak negative signals at shorter wavelengths, while the opposite is true of CMBF_d-Alt_. The Kuhn anisotropies of CMBF_d-Sor_, CMBF_l-Idi_ and CMBF_d-Alt_ are as strong as |*g*_390_| = 0.19, |*g*_380_| = 0.03, and |*g*_381_| = 0.32, respectively (Fig. 4b). TCD can be ascribed to both absorption-based OA (AOA) and scattering-based OA (SOA) (Fig. S7a†), which could be distinguished by diffuse reflectance UV-Vis (DRUV-Vis) and CD spectra with white (DRCD_W_)and black (DRCD_B_) backgrounds (Fig. S7b and c†).

With a white background, the DRCD_W_ spectra of CMBF_d-Sor_ and CMBF_l-Idi_ exhibit positive zero-crossing lines centered at 385 nm corresponding to the characteristic electron transition of semiconductor BiOBr,^41^ which might be dominantly contributed by the right-handedness (Fig. S8a†). With a black background, left-handedness in CMBF_d-Sor_ and CMBF_l-Idi_ preferentially reflects L-CP light and predominantly contributes to negative SOAs in the DRCD_B_ spectra and positive SOAs in the TCD spectra (Fig. S8b†). Both the DRCD_W_ and DRCD_B_ spectra of CMBF_d-Alt_ were opposite to those of CMBF_d-Sor_ and CMBF_l-Idi_, indicating the opposite handedness among them. The CD intensities might vary according to the structural chirality parameters induced by different DSAs. The constant TCD spectra obtained by rotating the sample stage and altering the tilting angle against the incident light of CMBF_d-Sor_ confirmed the absence of artifacts such as linear dichroism (Fig. S9†).

As expected, the racemic BiOBr films (Rc-BFs) and left-handed CMBFs were induced with six carbon DSAs of two mesomers (Gal (2*R*,3*S*,4*R*,5*S*) and All (2*R*,3*R*,4*S*,5*S*)), and d-Man (2*R*,3*R*,4*R*,5*R*), respectively (Fig. S10†). Additionally, the configurational effects of DSAs with different chain lengths and functional groups on the chirality of CMBFs were also investigated (not shown). The results also confirmed that the chirality of CMBFs is independent of the chirality and OAs of the DSA inducers. No chirality was exhibited in both the Rc-BF and achiral BiOBr film (A-BF) synthesized with an equal amount of d- and l-Sor and without using any symmetry breaking, respectively (Fig. S11 and S12†). The experimental results of post-added d-Sor to A-BP and A-BF, and synthesis of CMBFs with different d-Sor mass ratios confirm that the OA signals of CMBFs are induced by the chiral mesostructures of the chiral BiOBr rather than d-Sor molecules (Fig. S13 and S14†).

### Chirality transfer mechanism from diastereoisomers to CMBFs

DFT calculations and MD simulations were performed to clarify the chirality transfer mechanism from DSAs to CMBFs.^18,42,43^ The (001) monolayer crystalline surface of BiOBr was chosen as a basis model due to its exposed essence analyzed by TEM results. DFT calculations show that linear DSAs interact with BiOBr monolayers by weak physical adsorption rather than covalent bonds, and the linear adsorption pattern parallel to the (001) crystal plane and along the *X*- or *Y*-direction is the most stable state (Fig. S15†). The optimal adsorption patterns of DSAs on the upper and lower surfaces of the BiOBr monolayer are confirmed to be perpendicular to each other in periodic models (Fig. S16†).

**Fig. 5:**
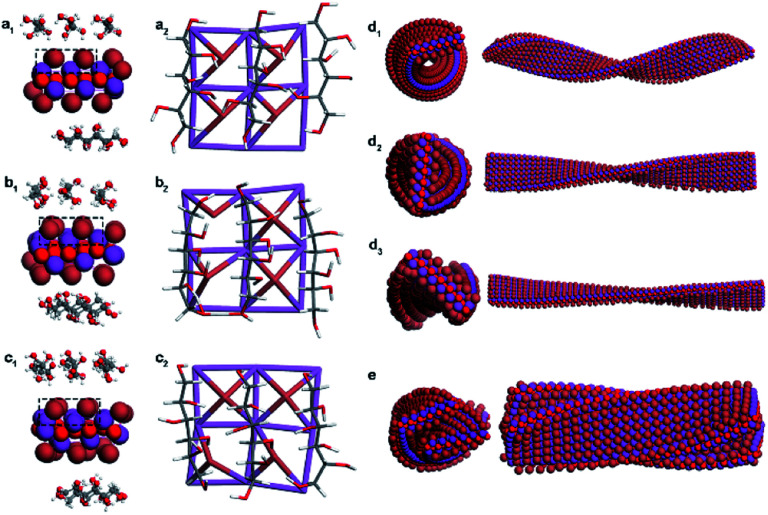
Asymmetric defects on the surfaces of BiOBr crystals induced by d-Sor (a), l-Idi (b), and d-Alt (c). MD simulations of the primary helical structure of BiOBr nanoflakes with different defect densities of 100% (d_1_), 67% (d_2_), and 33% (d_3_) in CMBF_d-Sor_, and their secondary stacking model (e).

In nonperiodic models that are closer to the nanoparticle phenomenon, the surface atoms of BiOBr induced with different DSAs undergo different degrees of distortion, especially for Br atoms on the surfaces (Fig. 5a and S17†). The original perfect Br–Bi tetragonal cone distorted to asymmetric centers, where four asymmetric defects with left-handedness were generated for CMBF_d-Sor_ (Fig. 5a), one left-handed chiral defect and racemic defects were found for CMBF_l-Idi_ (Fig. 5b), and two and three right-handed defects were found for CMBF_d-Alt_ and CMBF_d-Man_, respectively (Fig. 5c and S17c†). As expected, racemic defects with opposite handedness in pairs were found for CMBF_Gal_, and no defects were observed for CMBF_All_ (Fig. S17a and b†). It can be found that changing the conformation of the carbon chiral center at any one or two sites of the DSA molecule leads to the formation of different asymmetric defects on the BiOBr surface. These asymmetric defects would be the geometrical basis for triggering the chiral twists in the BiOBr monolayer structures.

Ligand-free models of BiOBr with a lateral size of 6 × 24 × 1 unit cells with a twisted crystal lattice were constructed using a universal force field (UFF). The BiOBr monolayer, which was originally a planar structure, rapidly underwent continuous twisting after 1 ns relaxation, and a left-handed helix was obtained for CMBF_d-Sor_ and CMBF_l-Idi_ (Fig. S18a and b†), and a right-handed helix was obtained for CMBF_d-Alt_ (Fig. S18c†), which were considered as the primary chirality of nanoflakes. The helical conformation of BiOBr nanoflakes with different lengths was constructed, and the increase in length led to a more complete periodic chiral helix structure (Fig. S19†).

Considering the weak physical absorption of DSAs on the BiOBr surface, the efficiency of triggering chiral distortion is expected to be very low. Therefore, we further investigated the effect of different densities of chiral defects on the helical BiOBr nanoflakes. The results showed that the tortuosity and helical pitch of the twisted nanoflakes can be effectively regulated by controlling the density of the chiral defects. Higher densities of chiral defects lead to higher tortuosity and a smaller pitch length of nanoflakes (Fig. 5d and S20†). MD simulations showed that the arrangement of twisted BiOBr nanoflakes was confirmed to be right-handed for CMBF_d-Sor_ and CMBF_l-Idi_, in contrast to CMBF_d-Alt_, which was considered as the secondary chirality (Fig. 5e, S20a_4_ and b_4_†). The simulations and experimental results displayed great consistency in this work. However, because of computational capacity and cost limitations, higher-level chiral structures consisting of large numbers of atoms are difficult to implement.

According to electron crystallography, CD spectra and theoretical calculations, the chirality of each level was determined. Three levels of chirality existed in the CMBFs: primary lattice distortion of an individual nanoflake, secondary chiral nanoplates formed by helical stacking of primary nanoflakes, and tertiary chiral vortexes arranged by secondary chiral nanoplates. A speculative possibility of OAs contributed by each level of chirality was given by peak differentiation simulation of CD and UV/Vis spectra based on the experimental results (Fig. S21, Tables S1 and S2†). It should be noted that this simulation is limited to the assumption that the AOAs and SOAs are based on the theory of the exciton couplet and circular Bragg resonance, respectively.

Therefore, the chiral center configuration of the DSA molecule plays an important role in determining the chirality of CMBFs. It determines the spatial geometrical matching of the DSA molecule and the BiOBr crystal surface, which further influences the multilevel chirality of BiOBr. Taking d-Sor with a configuration of (2*R*,3*R*,4*R*,5*S*) as an example, the absorption of d-Sor generated left-handed defects, which triggered the formation of left-handed primary BiOBr nanoflakes and right-handed secondary chiral arrangement of these primary nanoflakes. l-Idi with a configuration of (2*R*,3*R*,4*R*,5*S*) by changing the configuration of carbon 2 induced left-handed defects, which triggered the similar chirality as the d-Sor. Changing the configuration of carbons 3 and 4, the mesomeric Gal and All with a configuration of (2*R*,3*S*,4*R*,5*S*) and (2*R*,3*R*,4*S*,5*S*) were obtained, respectively. No chirality was formed. However, the d-Man with the configuration of (2*R*,3*R*,4*R*,5*R*) by changing the configuration of carbon 5 induced the formation of right-handed defects, which triggered the formation of right-handed primary BiOBr nanoflakes and left-handed secondary chiral arrangement of these primary nanoflakes, and this phenomenon could also be observed in the d-Alt (2*R*,3*S*,4*R*,5*R*) by changing the configuration of both carbons 3 and 5. Changing the conformation of any other two or three carbon chiral centres in a sugar alcohol molecule and the resulting molecule is enantiomerically identical to the molecule mentioned above.

By summarizing the laws, it was found that the chirality of CMBFs was dependent on the geometric matching of BiOBr and DSA molecules at the adsorption interface rather than the DSA stereochemistry. When the hydroxyl groups at the positions of carbon atoms 3, 4 and 5 in the DSA molecule are aligned parallelly on the surfaces of BiOBr, left-handed primary chiral BiOBr nanoflakes are formed, while when the hydroxyl groups at the positions of carbon atoms 2, 3 and 5 are aligned anti-parallelly, right-handed primary chirality is formed. The primary chiral BiOBr nanoflakes are arranged in an opposite chiral arrangement to form the secondary chiral nanoplate. The tertiary chirality of CMBFs is induced by the chiral arrangements of DSA molecules under hydrothermal conditions. Therefore, the chiral molecules with multiple chiral centers could be used as symmetry-breaking agents to induce the desired chirality of materials by changing the configurations of the chiral centers. However, this principle may be different for different synthetic systems and materials, depending on the interactions of the material with the chosen chiral molecules.

## Conclusions

To the best of our knowledge, this is the first report describing chirality transfer from diastereoisomers to achiral inorganic species. The theoretical calculations reveal that the geometric matching and chiral defect densities could affect the tortuosity and pitches of the assembly of achiral inorganic nanoparticles. These insights not only provide a promising strategy for fabricating chiral inorganic nanostructures but also provide valuable information for understanding the origin of homochirality in organisms.

## Data availability

Raw data are available upon request from the authors.

## Author contributions

K. D. carried out the experimental synthesis and SEM, XRD, and CD characterization studies; J. A. conducted the TEM measurement; Z. Q performed DFT calculations and MD simulations; K. D. is responsible for all data processing and creating the original draft and revising the manuscript under the supervision of Y. D., L. H., Z. Q., and S. C.

## Conflicts of interest

There are no conflicts to declare.

## Supplementary Material

SC-013-D1SC05601H-s001
