# Supplementary material for: Mechanism of diastereoisomer-induced chirality of BiOBr
Source: Chem Sci. 2022 Feb 4;13(8):2450–5. doi: 10.1039/d1sc05601h (PMC8864704; doi:10.1039/d1sc05601h)
Supplement: SC-013-D1SC05601H-s001 [file SC-013-D1SC05601H-s001.pdf]

Electronic Supplementary Material (ESI) for Chemical Science.
This journal is © The Royal Society of Chemistry 2022

Electronic Supplementary Material (ESI) for Chemical Science.
This journal is © The Royal Society of Chemistry 2021

Supporting Information

Mechanism of diastereoisomer-induced chirality of BiOBr

Kun Ding[a†], Jing Ai[b†], Yingying Duan[b*], Lu Han[b*], Zhibei Qu[a,c*] and Shunai Che[a,b*]

Contents

1. Experimental Methods 2
   1.1 Chemicals..2
   1.2 Substrate activation...2
   1.3 Synthesis of CMBFs... ...2
   1.4 Characterization...2
   1.5 DFT calculations and MD simulations.......................................2
2. Supplementary Figures and Tables 3-16
3. References 17

1. Experimental Methods

1.1 Chemicals

Bismuth nitrate pentahydrate ($Bi(NO_3)_3•5H_2O$) was purchased from Macklin. Sodium bromide (NaBr) and ethylene glycol (EG) were purchased from Sinopharm Chemical Reagent. D-sorbitol (D-Sor), L-iditol (L-Idi), allitol (All), D-altritol (D-Alt) and galactitol (Gal) were purchased from TCI. D-mannitol (D-Man) was provided by Adamas. The deionized (DI) water (resistivity ~18.2 MΩcm) was obtained from a Milli-Q synthesis system. All reagents were used without further purification.

1.2 Substrate activation

The FTO substrates (40×10×1.1 mm, coating thickness = 500 nm, sheet resistance ≤ 15 Ω) were washed with acetone, ethanol and deionized water for 15 min respectively in an ultrasonic bath, followed by soaking in the 1 mM $Bi(NO_3)_3•5H_2O$ nitric acid solution for 24 h, and then dried at 80 °C.

1.3 Synthesis of CMBFs

The CMBFs were prepared by diastereomers-directed hydrothermal method. Typically, 0.5 mmol diastereomeric sugar alcohol (DSA) was dissolved in a mixed solution of 4 mL of H_2O and 16 mL of EG with stirring for 20 min, and then, 0.5 mmol $Bi(NO_3)_3$•$5H_2O$ was added to obtain a homogeneous solution. After the mixture was stirred for 20 min, 0.5 mmol NaBr was added, followed by adding 75 µL NaOH (0.1M). After stirring another 20 min, the above mixed solution and activated FTO substrate were transferred into a 50 ml Teflon-lined autoclave, and hydrothermally heated at 140 °C for 24 h. After cooling to room temperature, the FTO substrate coated with BiOBr was washed with DI water and anhydrous ethanol for several times to remove the unreacted impurities, and then dried at 80 °C for 12 h.

We investigated the effects of configuration of carbon chiral centers of DSAs (such as RRRS for D-Sor, SRRS for L-Idi, RSRS for Gal, RRSS for All, RRRR for D-Man, RSRR for D-Alt) on the resulting morphology and optical activity (OA) of CMBFs. It should be noted that the synthetic molar composition of all the CMBFs was 0.5 DSA: 0.5 $Bi(NO_3)_3$•$5H_2O$: 0.5 NaBr: 287 EG: 222 H_2O: 7.5×10^{-4} NaOH.

1.4 Characterization

SEM images were obtained using JEOL JSM-7900 with an accelerating voltage of 5.0 kV and GBSH mode. TEM images were taken with an JEOL JEM-F200 TEM microscope operated at 200 kV. Images were recorded using a Gatan OneView IS camera. A thin section preparation was performed after the mixing the BiOBr powder peeled off from the CMBFs with an epoxy resin and further solidifying. The section with thicknesses of 30-40 nm was prepared on a Leica EM UC7 Ultramicrotome with a diamond knife, which was then loaded onto TEM grid. Powder X-ray diffraction patterns were recorded on a Bruker Advance 8 equipped with Cu Kα radiation (40 kV, 20 mA). The ^{13}C CP/MAS NMR spectrum was recorded using 600 M AVANCE NEO solid-state NMR spectrometer at the resonance frequency of 150 MHz with 3.2 mm of ZrO_2 rotor and a spinning speed of VR = 15.0 kHz, a 0.49s excitation pulse, a 4s relaxation delay, and 900 scans. Fourier-transform infrared spectra were recorded on a Bruker ALPHA spectrophotometer in the region of 4000~400 cm^{-1} with a resolution of 2 cm^{-1}. Raman spectra were recorded using DXR Raman spectrometer with 532 nm laser irradiation. Thermogravimetry (TG) analysis was carried out on a Thermogravimetric Analyzer Q5000IR with heating rate of 10 ℃ min^{-1} and air atmosphere. The DRCD and TCD spectra were taken by using a JASCO J-1500 spectropolarimeter fitted with DRCD apparatus.

1.5 DFT calculations and MD simulations

All first-principles calculations were carried out using the VASP program[1], where the Kohn–Sham equations were solved by means of the Perdew, Burke, and Ernzerhof exchange–correlation functional[2] with the projector-augmented-wave (PAW) pseudopotentials[3]. A correction to the PBE wave function was made (PBE + U) by including a repulsive on-site Coulomb interaction, *U*[4]. The orbital dependence of the Coulomb and exchange interactions has been taken into account with this scheme, using a value of 6 eV for the Hubbard parameter for Bi[5]. The plane-wave functional was using a cutoff-energy of 520 eV and the Brillouin zones were sampled through Monkhorst–Pack special k-point grids[6] that ensured geometrical and energetic convergence for BiOBr structures and polyols' absorption in this work.

MD simulations using a classical force field were used to model nanosheets comprising BiOBr nanosheets with specified chiral defects from above DFT calculations. It should be noted that chiral ligands were removed from the models considering the relatively weak physical absorption of polyols, verified by our experiments. The MD simulations were performed with Open Babel toolbox[7], using the universal force field (UFF) to treat bonding and nonbonding interactions[8] in the models. The Coulombic force was calculated atom-based with a distance cutoff of 1.55 nm. NVT ensemble was used at the ambient temperature of 298K, controlled by the Nosé method[9]. A time step of 1 fs was employed while the total production time was set up to 10 ns before 1 ns pre-equilibrium.

2. Supplementary Figures and Tables

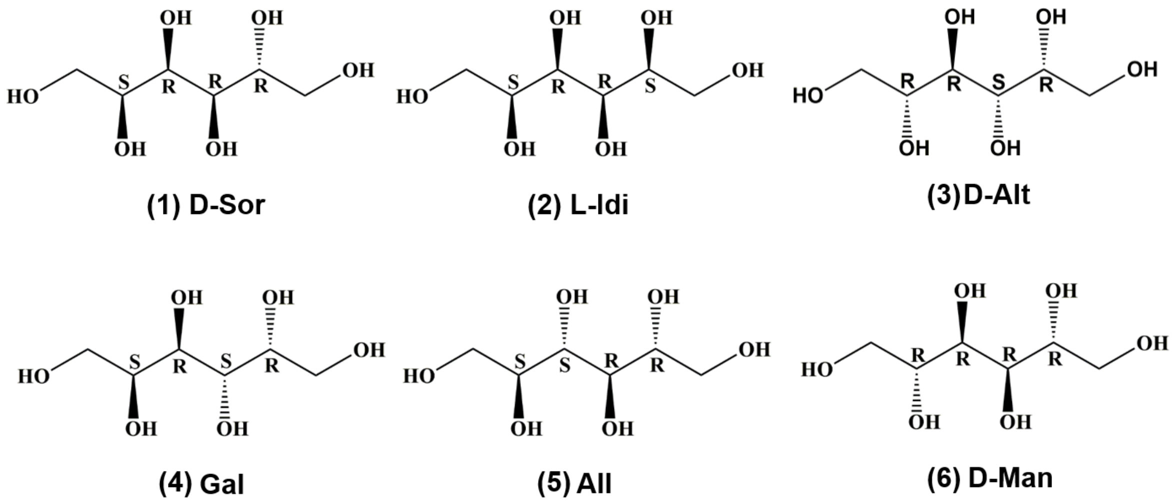

Figure S1. Configurations of DSAs with six carbons and four chiral centers.

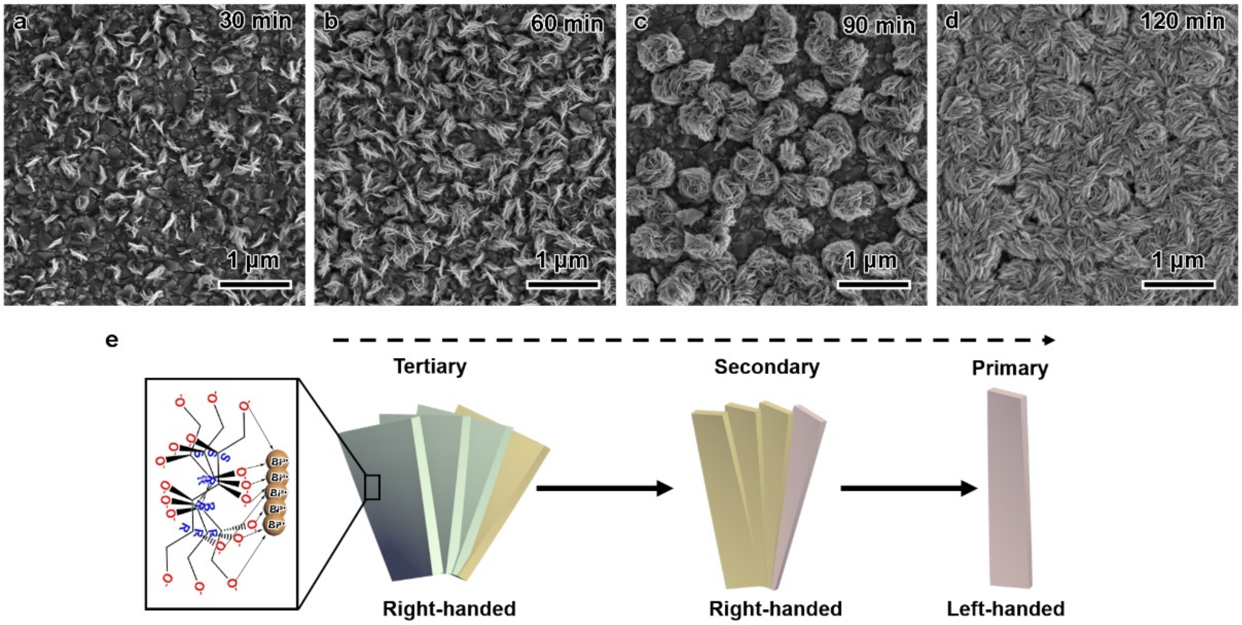

Figure S2. SEM images of $CMBF_{D\text{-}Sor}$ sampled at different reaction times of 30 min (a), 60 min (b), 90 min (c), 120 min (d), as well as the schematic diagram of the chiral assembly process in hierarchical chiral structures (e).

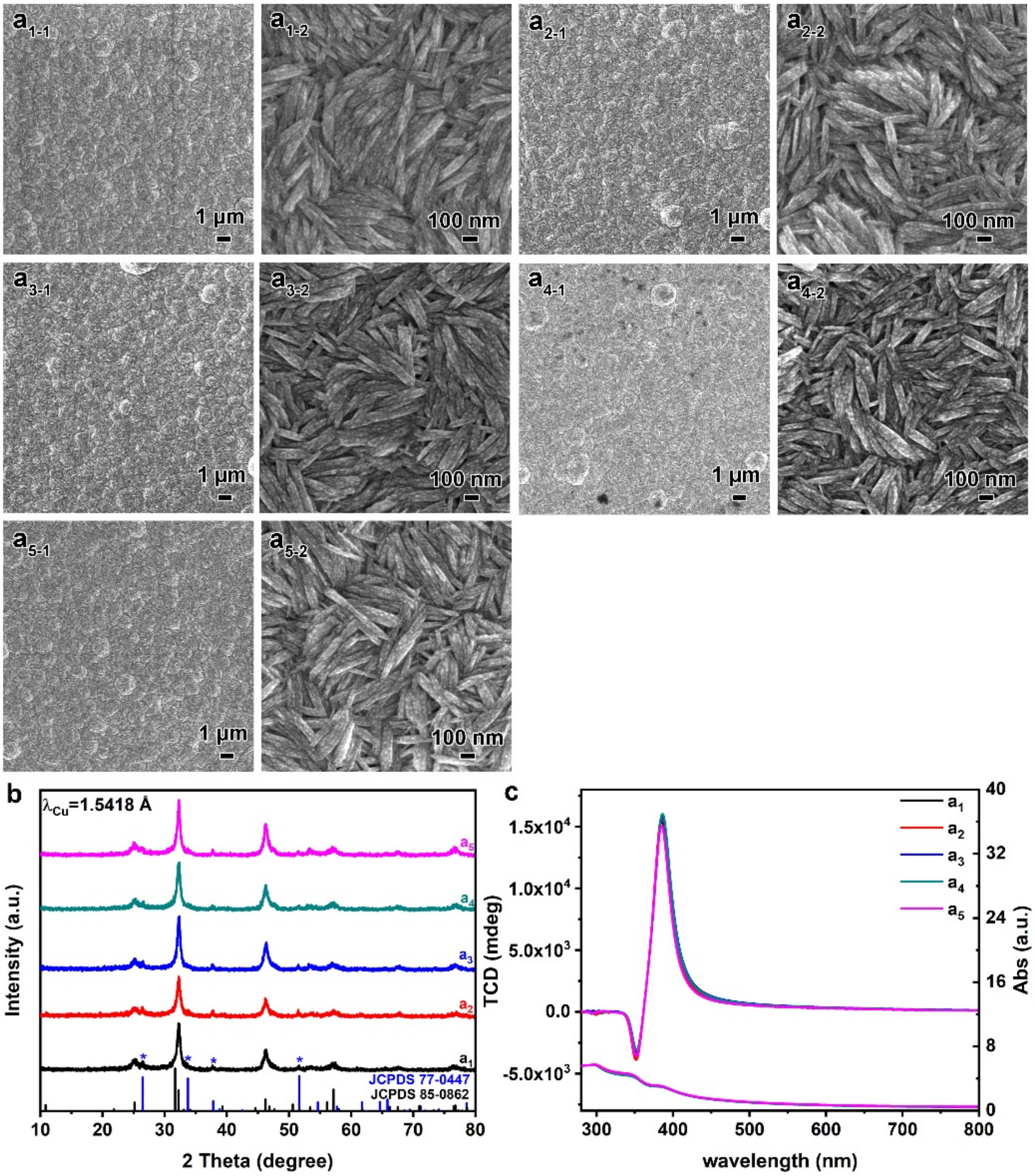

Figure S3. SEM images (a), XRD patterns (b), and TUV-Vis and TCD spectra (c) of the repeated $CMBF_{D\text{-}Sor}$.

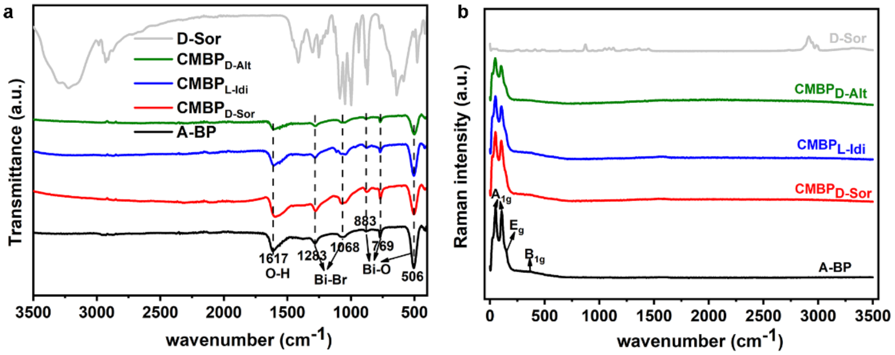

Figure S4. FTIR (a) and Raman (b) spectra of achiral BiOBr powder (A-BP), $CMBP_{D\text{-}Sor}$, $CMBP_{L\text{-}Idi}$, $CMBP_{D\text{-}Alt}$, and D-Sor. The A-BP, $CMBP_{D\text{-}Sor}$, $CMBP_{L\text{-}Idi,}$ and $CMBP_{D\text{-}Alt}$ were scraped from achiral BiOBr film (A-BF), $CMBF_{D\text{-}Sor}$, $CMBF_{L\text{-}Idi,}$ and $CMBF_{D\text{-}Alt}$, respectively. Achiral BiOBr was synthesized without using any DSA.

As shown in Figure S4, the FTIR and Raman spectra of CMBFs synthesized with different DSAs exhibit the identical peaks as A-BP, and the peaks of DSA was not observed, indicating the absence of DSAs in CMBFs.

The peak at 1617 cm^{-1} is attributed to the flexural vibration of the O-H in chemisorbed and/or physically absorbed H_2O, the peaks at 1283 cm^{-1} and 1068 cm^{-1} could be assigned to the asymmetric and symmetric stretching vibration of Bi-Br, the peaks below 1000 cm^{-1} belong to the typical stretching vibrational modes of Bi-O [10, 11].

As shown in the Raman spectra of the samples (Figure S4b), the strong bands at 52 cm^{-1} and 110 cm^{-1} could be assigned to A_{1g} external and internal stretching modes of Bi–Br, the band at 160 cm^{-1} is ascribed to the E_g internal Bi–Br stretching mode, and the band at 372 cm^{-1} is assigned to the B_{1g} mode involving motion of the oxygen atoms [12].

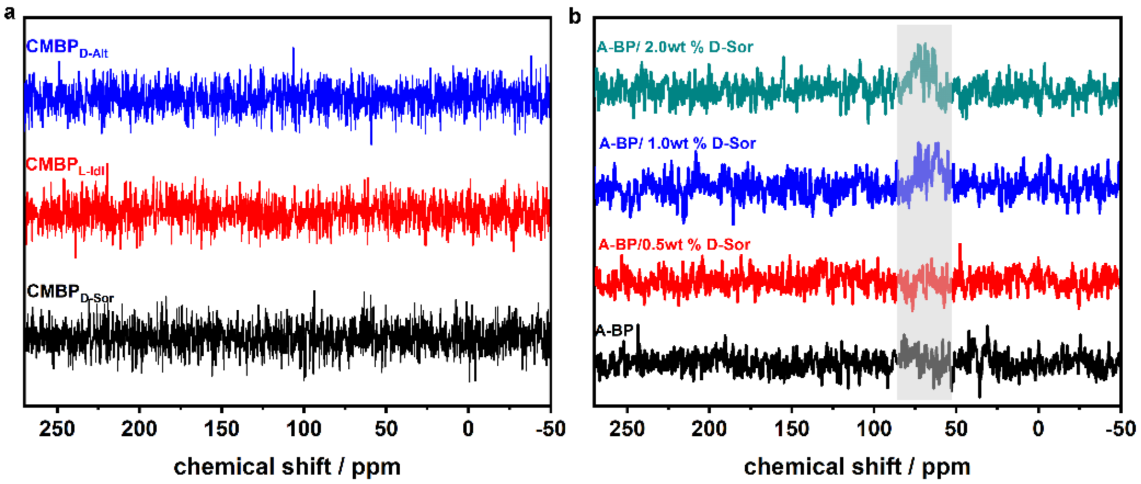

Figure S5. Solid-state ^{13}C CP/MAS NMR spectra of CMBP$_{D-Sor}$, CMBP$_{L-Idi}$, and CMBP$_{D-Alt}$ (a), as well as the A-BP/D-Sor with different mass ratios with D-Sor (b). The gray shaded part represents the chemical shift of C in D-Sor molecule.

As shown in Figure S5a, no peaks of any carbon were observed in the NMR spectra of CMBF$_{D-Sor}$, CMBF$_{L-Idi}$, and CMBF$_{D-Alt}$, indicating the absence of DSAs in CMBFs.

In addition, 80 mg of achiral BiOBr powder was mixed with D-Sor with different mass ratios, and thoroughly ground with a mortar. After the mixture was well mixed, the ^{13}C CP/MAS NMR spectra of samples were tested. As shown in Figure S5b, the extremely weak peak could be detected when the mass ratio of D-Sor molecule was 0.5 wt %. As the D-Sor increases, the peak gradually becomes stronger. No C peaks were observed in CMBFs, indicating that the DSA residue in CMBFs is less than 0.5 wt %.

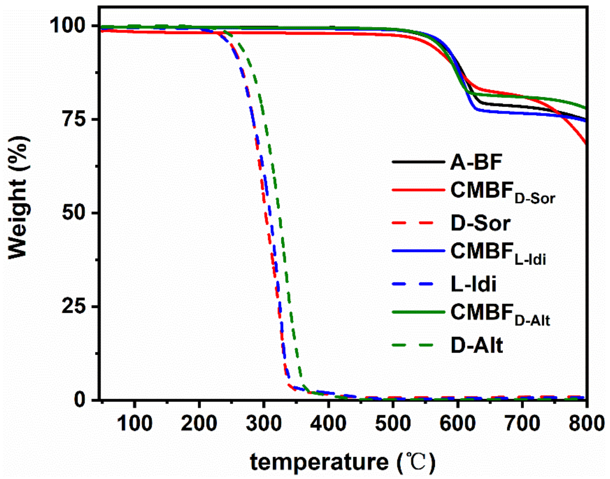

Figure S6. TG curves of CMBFs synthesized with different DSAs (solid lines) and DSAs alone (dash lines).

Figure S6 shows the TG curves of CMBFs, A-BF, and DSAs. The DSAs molecules began to decompose at 200 °C and were almost completely decomposed at 350 °C. Both A-BF and CMBFs decomposed from 500 °C due to the instability of Br. No obvious weight loss was observed before 350 °C for both A-BF and CMBFs, indicating the absence of organics in samples.

Since the DSA molecules have high solubility in water and they are adsorbed on the BiOBr surface by weak physical adsorption. Thus, the results of FTIR, Raman, NMR and TG analysis were able to confirm that the DSAs in CMBFs have been completely removed.

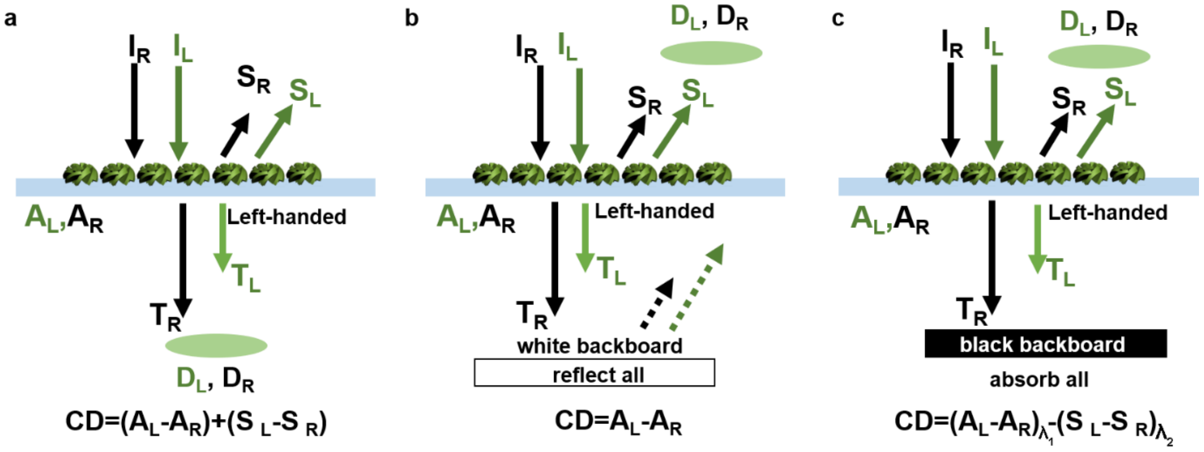

Figure S7. Schematic illustration of CD detection mechanism with a left-handed medium: TCD spectrum (a), $DRCD_W$ (b), and $DRCD_B$ (c) spectra. Black and green arrows represent to right- and left-handed circularly polarized (R- and L-CP) light, respectively. I is the incident linearly unpolarized light (I_L=I_R); A, T, S, and D are absorbed, transmitted, scattered, and detected CP light, respectively. In all of test modes, $I_L= A_L+S_L+T_L$, $I_R= A_R+S_R+T_R$.

Figure S7 shows the different detection modes of UV/Vis and CD spectra: (i) TUV/Vis and TCD mode; DRUV/Vis and (ii) $DRCD_W$ and (iii) $DRCD_B$ modes.

In TCD mode:

(i) The incident light from light source is I_L+I_R ($I_L=I_R$).

(ii) The light absorbed by CMBF is A_L+A_R ($A_L\neq A_R$), the light scattered by CMBF is S_L+S_R ($S_L\neq S_R$), the remaining light transmitted through CMBF is T_L+T_R ($T_L\neq T_R$).

(iii) The light collected by the detector is D_L+D_R ($D_L=T_L$, $D_R=T_R$).

(iv) The output TCD signal is I_{total} - D_{total}.

Thus, TCD=I_{total} - D_{total}=$(I_L+I_R)-(D_L+D_R)=(I_L-D_L)-(I_R-D_R)=(A_L+S_L)-(A_R+ S_R)=(A_L-A_R)+(S_L-S_R)$, which includes the absorption-based optical activity (AOA) and scattering-based optical activity (SOA).

In this case, TUV/Vis is the extinction caused by absorption and scattering.

In $DRCD_W$ mode:

(i) The incident light from light source is I_L+I_R ($I_L=I_R$).

(ii) The light absorbed by CMBF is A_L+A_R ($A_L\neq A_R$), the light scattered by CMBF is S_L+S_R ($S_L\neq S_R$), the remaining light transmitted through CMBF is T_L+T_R ($T_L\neq T_R$).

(iii) With a white background, the transmitted light at visible range is totally reflected.

(iv) The light collected by the detector is D_L+D_R ($D_L=T_L+S_L$, $D_R=T_R+S_R$). Specifically, in DRCD mode, the scattered light is collected by an integrating sphere, so all the scattered and background-reflected light can be collected into the detector.

(v) The output $DRCD_W$ signal is I_{total} - D_{total}.

Thus, $DRCD_W=I_{total}$ - D_{total}=$(I_L+I_R)-(D_L+D_R)$= $(I_L-D_L)-(I_R-D_R)$= $(I_L-T_L-S_L)-(I_R-T_R-S_R)=A_L-A_R$, which was AOA.

In this case, DRUV/Vis-$_W$ is the extinction caused by absorption.

In $DRCD_B$ mode:

(i) The incident light from light source is I_L+I_R ($I_L=I_R$).

(ii) The light absorbed by CMBF were A_L+A_R ($A_L\neq A_R$), the light scattered by CMBF is S_L+S_R ($S_L\neq S_R$), the remaining light transmitted through CMBF is T_L+T_R ($T_L\neq T_R$).

(iii) With a black background, the transmitted light at visible range is totally absorbed.

(iv) The light collected by the detector is D_L+D_R ($D_L=S_L$, $D_R=S_R$).

(v) The output $DRCD_B$ signal is I_{total} - D_{total}.

In this case, there are two conditions: If the resonance wavelengths of absorption and scattering are the same, the $DRCD_B = I_{total} - D_{total} = (I_L+I_R)-(D_L+D_R) = (I_L-D_L)-(I_R-D_R) = (I_L-I_R)-(S_L-S_R) = -(S_L-S_R)$, which exhibits SOA only; If the materials exhibit AOA across the entire UV/Vis absorption band, $DRCD_B=(A_L-A_R)_{\lambda 1}-(S_L-S_R)_{\lambda 2}$, where the λ_1 represent the absorption band at UV/Vis range, λ_2 represent the absorption band at visible range.
In this case, DRUV/Vis-$_B$ is the extinction caused by absorption and scattering.

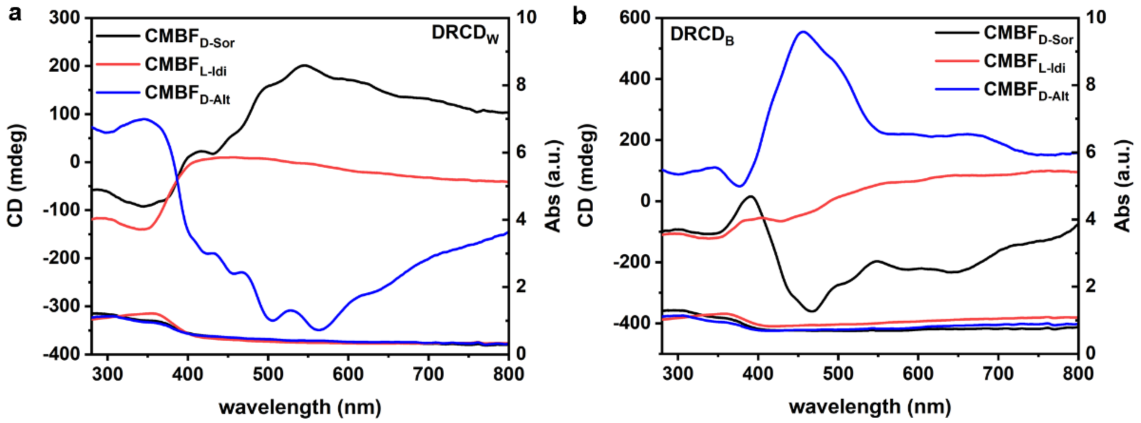

Figure S8. DRUV–vis and DRCD spectra of CMBFs synthesized with different DSAs, measured with a white (a) and black (b) background.

With a white background, the DRCD spectrum of CMBF$_{D\text{-}Sor}$ and CMBF$_{L\text{-}Idi}$ exhibit positive CD signals in the range of 360-800 nm and negative CD signals in 280-360 nm (Figure S8a), indicating that the CMBF$_{D\text{-}Sor}$ and CMBF$_{L\text{-}Idi}$ preferentially allow the absorption of left-handed CP (L-CP) light in 360-800 nm, and *vice versa* in the 280-360 nm. With a black background, the DRCD spectra exhibit CD signals that are opposite to the TCD and DRCD with a white background, indicating that the CMBF$_{D\text{-}Sor}$ and CMBF$_{L\text{-}Idi}$ preferentially allow the reflection of L-CP light due to the predominant effect of the left-handed chiral structure, and *vice versa* in the CMBF$_{D\text{-}Alt}$, in which the right-handed chiral structure dominantly contributed to the OAs (Figure S8b).

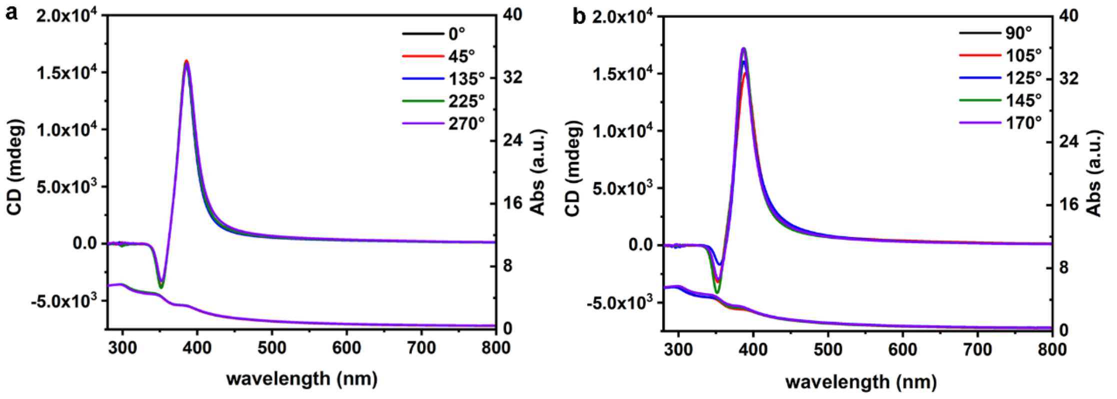

Figure S9. TCD spectra of CMBF$_{D\text{-}Sor}$ at different angles by rotating the sample (a) and with different tilting angles of the film against the incident light (b).

TCD spectra of CMBF$_{D\text{-}Sor}$ were detected by rotating sample stage (Figure S9a), no obvious changes were observed, indicating the absence of linear dichroism in the TCD results. Furthermore, the TCD spectra of CMBF$_{D\text{-}Sor}$ with different tilting angle against the incident light were detected, the weakly blue-shifted CD signals at wavelengths of 280-800 nm indicate that the chiral axis of primary chirality is dominantly perpendicular to the substrate and the SOA exists across the UV-Vis bands (Figure S9b).

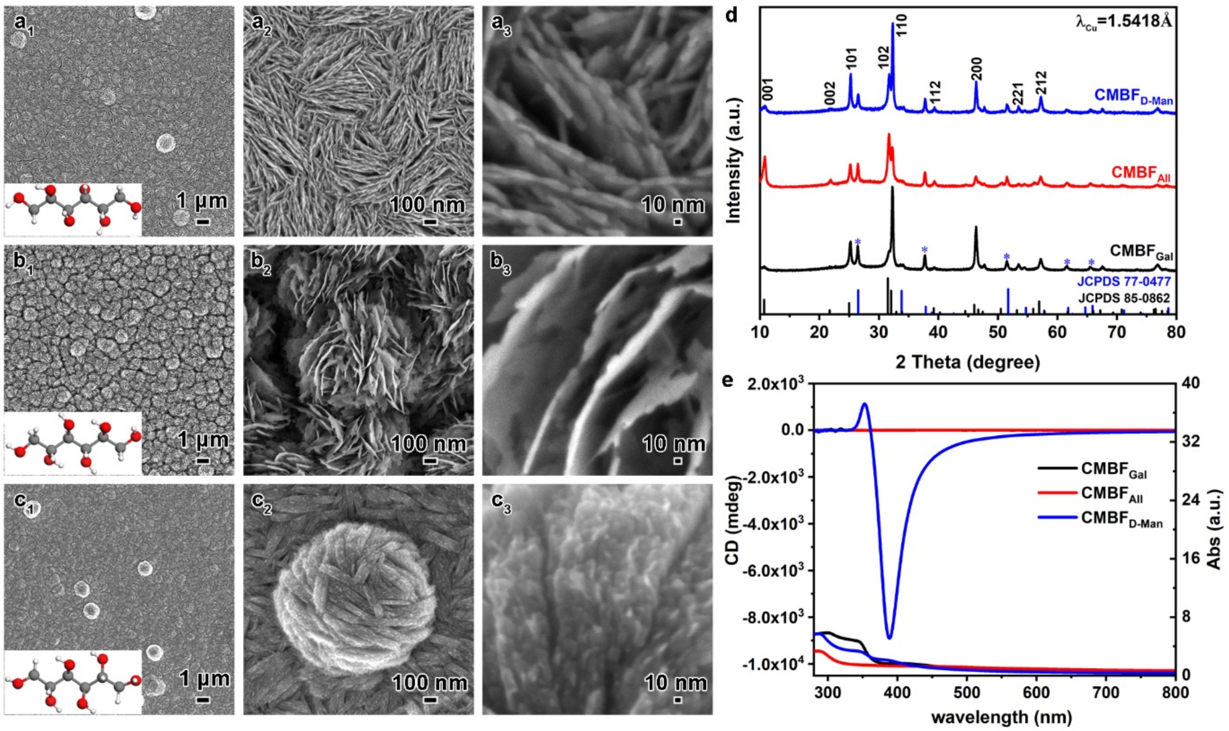

Figure S10. SEM images (a-c), XRD patterns (d), and TCD spectra (e) of CMBFs synthesized using DSAs with six carbons and four chiral centers with different configurations as symmetry-breaking agents: (a_1-a_3) Gal with configuration of (2R,3S,4R,5S), (b_1-b_3) All with configuration of (2R,3R,4S,5S), (c_1-c_3) D-Man with configuration of (2R,3R,4R,5R).

Gal (2R,3S,4R,5S) and All (2R,3R,4S,5S) are mesomers, in which the configuration of carbon 3 and carbon 4 of D-Sor (2R,3R,4R,5S) were changed (insets of Figures S10a_1, and b_1). The morphologies of the $CMBF_{Gal}$ and $CMBF_{All}$ changed significantly, and their chiralities degraded completely (Figures S10a and b).

D-Man (2R,3R,4R,5R) was resulted by changing the configuration of carbon 5 of D-Sor (2R,3R,4R,5S). The $CMBF_{D-Man}$ composed of nanoplates with lengths of 150-550 nm and thicknesses of 20-100 nm arranged in a counter-clockwise manner was observed (Figure S10c_2). Furthermore, the nanoplates were formed by several rows of nanoflakes with thickness smaller than 10 nm (Figure S10c_3).

As shown in Figure S10d, the reflections of all samples can be well indexed to the pure tetragonal phase of BiOBr (JCPDS No. 85-0862). Notably, the intensities of 001 and 102 reflections of $CMBF_{All}$ is relatively enhanced, while the 200 reflection is weakened (red line in Figure S10d). The variation of the reflection intensities indicates that the configurations of carbon 2 and 4 in DSA are important in regulating the growth of CMBFs. The $CMBF_{Gal}$ and $CMBF_{D-Man}$ exhibit the similar growth orientation to the $CMBF_{D-Sor}$, suggesting that the chiral configurations of the carbon 3 and 5 do not affect their growth orientation but change their chiral morphologies.

Figure S10e shows the TCD spectra of CMBFs, a negative CD signal was observed for $CMBF_{D-Man}$ as for $CMBF_{D-Alt}$ (blue line in Figure 4), while no CD signals were found in $CMBF_{Gal}$ and $CMBF_{All}$. This result shows that the configurations of each carbon chiral center in the DSAs plays an important role in determining the chirality and OAs of resulting films.

The chirality of D-Man is the same to the D-Alt, consequently, the chirality of $CMBF_{D-Man}$ (Figure S10c) and $CMBF_{D-Alt}$ (Figure 3c) is the same. The results of theoretical calculations also confirmed that both $CMBF_{D-Alt}$ and $CMBF_{D-Man}$ produced right-handed defects (*vide post*).

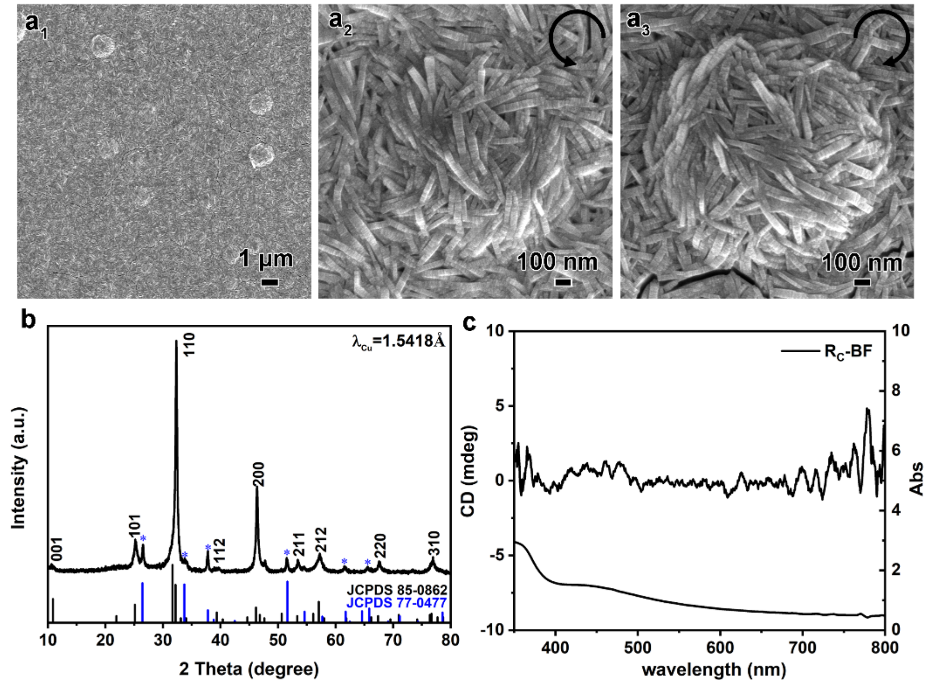

Figure S11. SEM images (a), XRD pattern (b), TUV/Vis and TCD spectrum (c) of Rc-BFs synthesized with racemic Sor molecule.

Figure S11 shows the Rc-BFs synthesized with equal amounts of D-Sor and L-Sor molecules. Both left- and right-handed nanoparticles were observed, but no CD signal was detected, indicating that there are equal amounts of left- and right-handed structures in the Rc-BFs, and the OA caused by them cancel each other. Ultimately, the film exhibits no chirality.

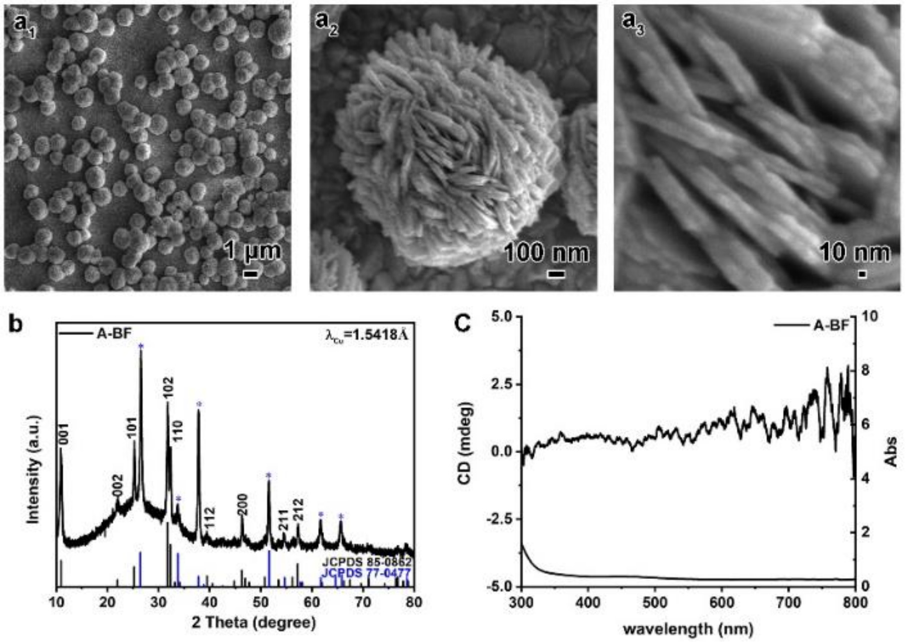

Figure S12. SEM images (a), XRD pattern (b), TUV/Vis and TCD spectrum (c) of A-BF synthesized in the absence of DSA molecule.

As shown in Figure S12, sparse A-BFs consisting of many nanoparticles was obtained in the absence of any symmetry-breaking agents. These nanoparticles are assembled from many nanosheets with disordered arrangements. As expected, the resulting A-BFs did not contain any chiral structure and no CD signal was detected because no chiral source was introduced during the synthesis.

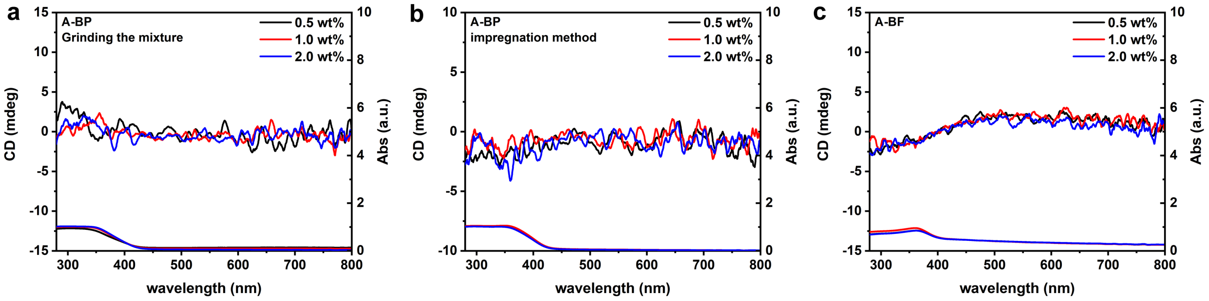

Figure S13. DRUV-Vis and DRCD spectra of A-BP mixed with different amounts of D-Sor by mechanical grinding (a) and impregnation (b). DRUV-Vis and DRCD spectra of A-BF impregnated with different amounts of D-Sor (c).

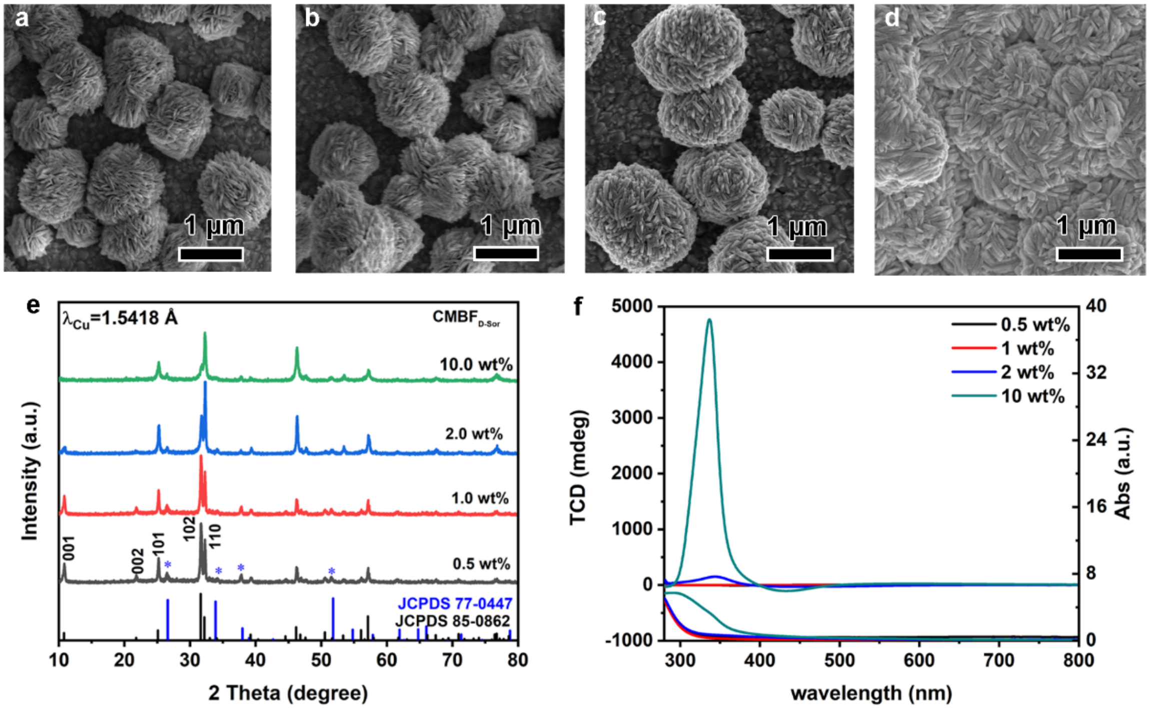

Figure S14. TUV-Vis and TCD spectra of CMBFs synthesized with different amounts of D-Sor.

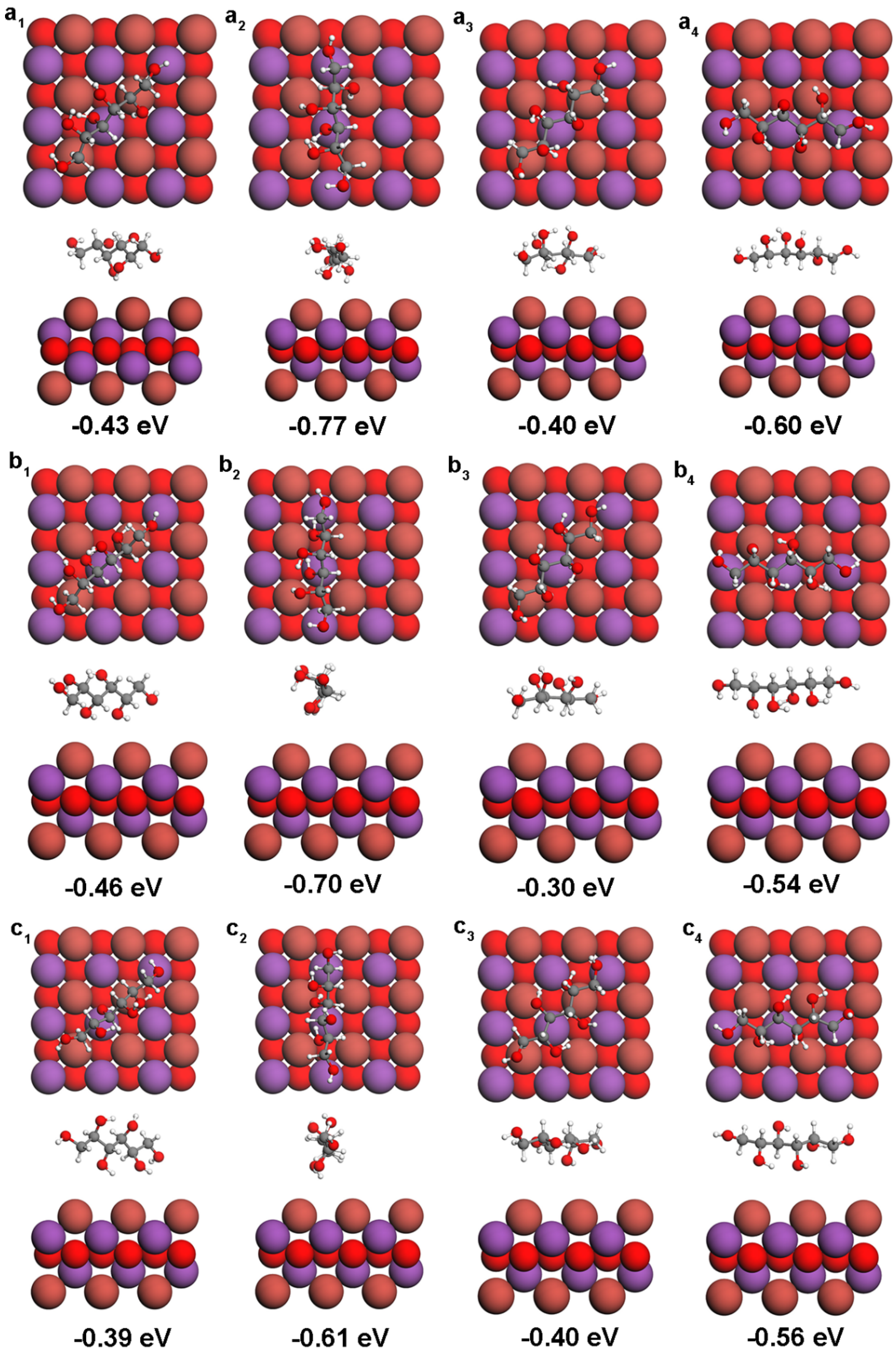

Figure S15. DFT calculations of possible adsorption orientations for D-Sor (a), L-Idi (b), and D-Alt (c) on the BiOBr (001) crystalline surface.

The adsorption energy values indicate the calculated relative stability with respect to the corresponding adsorption orientations. This result suggests a consistent adsorption pattern of DSAs on the BiOBr crystal surface.

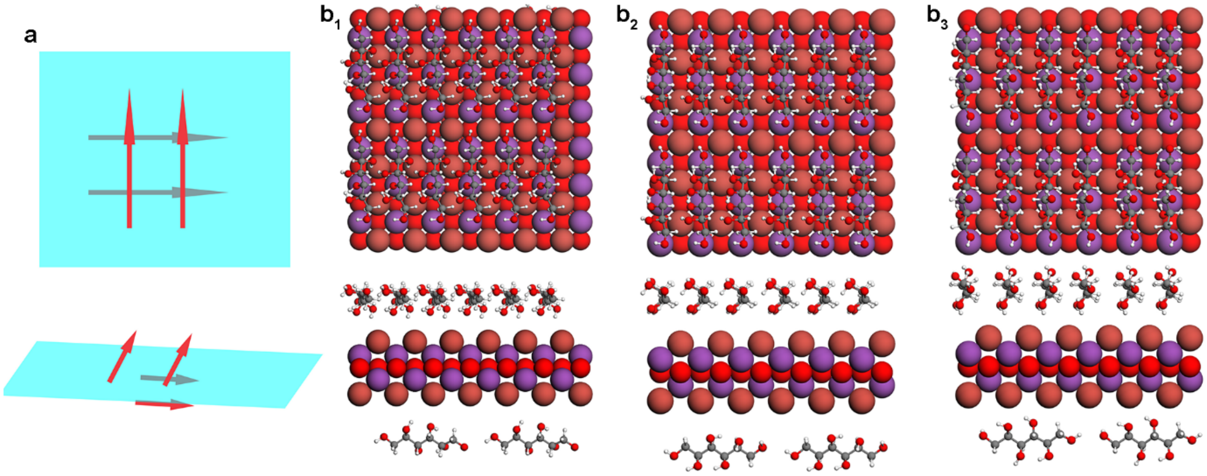

Figure S16. Schematic diagrams of the arrangement of DSA molecules (a), and the corresponding optimized structural models of D-Sor (b_1), L-Idi (b_2), and D-Alt (b_3) on the surfaces of BiOBr.

As shown in Figure S16a, the DSAs are adsorbed on the upper and lower surfaces of BiOBr monolayer with perpendicular orientation to each other, indicating that the helix caused by the DSAs is oriented along an angle of 45°.

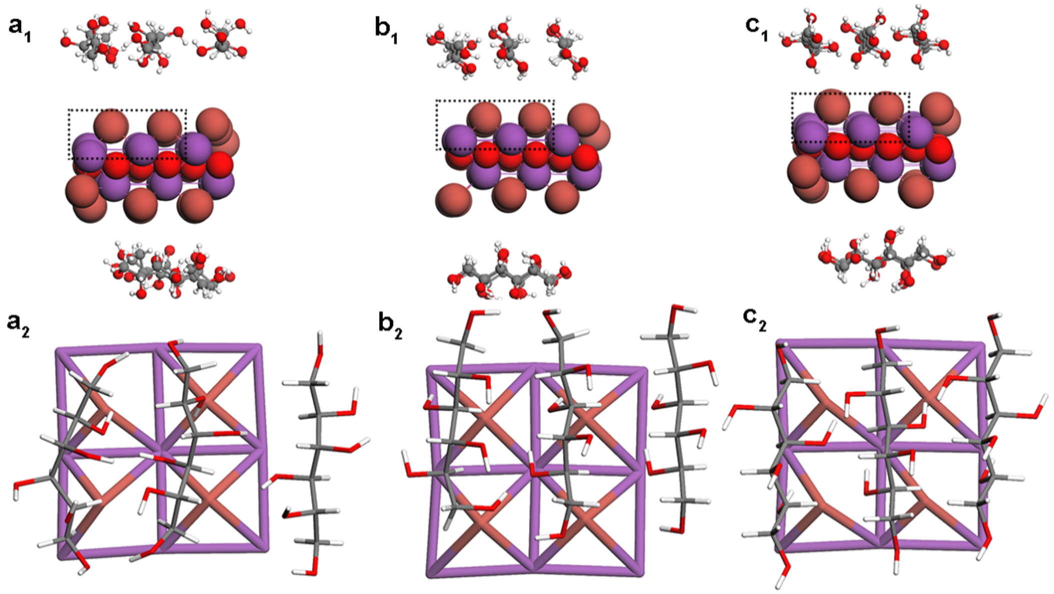

Figure S17. Asymmetric defects formed in $CMBF_{Gal}$ (a), $CMBF_{All}$ (b), and $CMBF_{D\text{-}Man}$ (c).

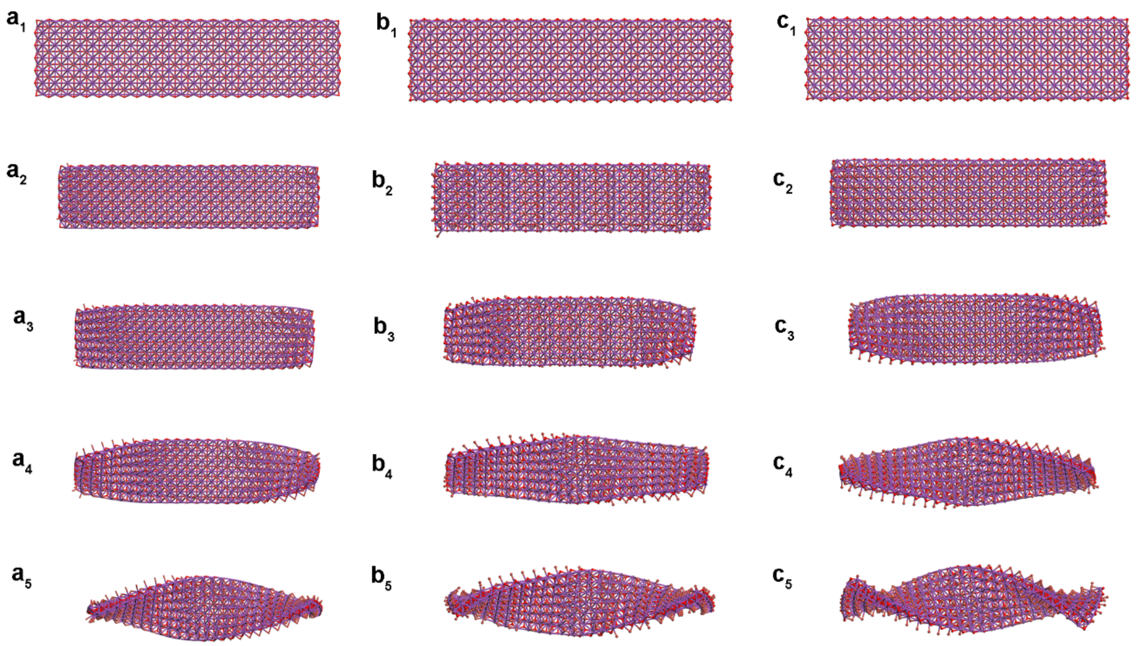

Figure S18. The evolution process of primary chirality induced by the asymmetric defects in $CMBF_{D\text{-}Sor}$ (a), $CMBF_{L\text{-}Idi}$ (b), and $CMBF_{D\text{-}Alt}$ (c).

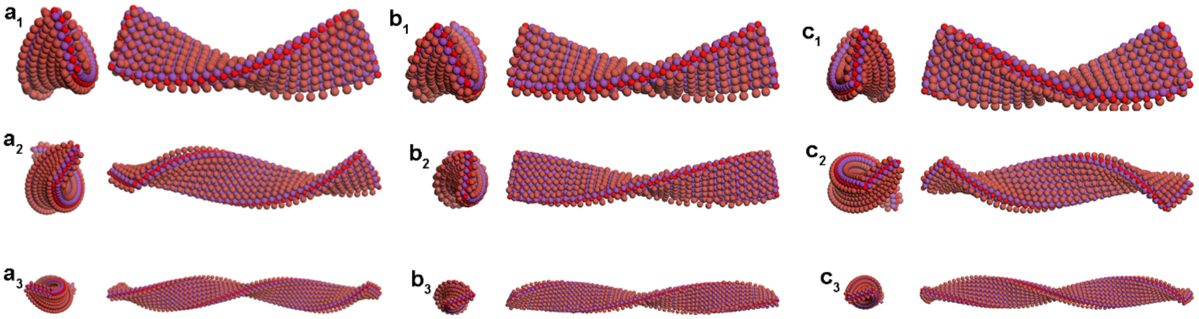

Figure S19. The primary chirality with different twist lengths of 6×24(a_1, b_1, c_1), 6×36 (a_2, b_2, c_2), and 6×60 (a_3, b_3, c_3) in $CMBF_{D\text{-}Sor}$ (a), $CMBF_{L\text{-}Idi}$ (b), and $CMBF_{D\text{-}Alt}$ (c).

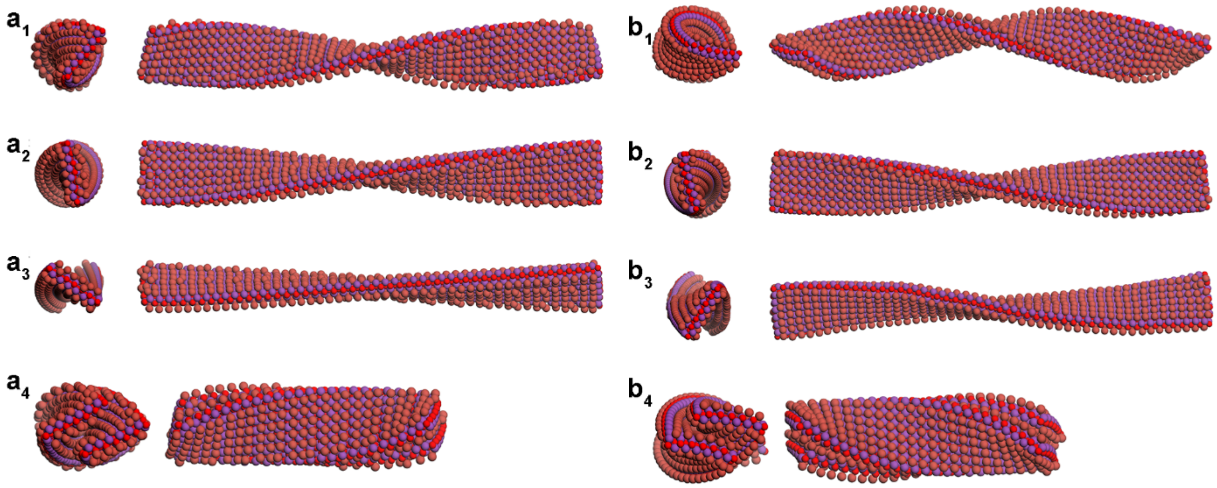

Figure S20. MD simulations of primary helical structures of BiOBr nanoflakes with different defect densities of 100 % (a_1, b_1), 67% (a_2, b_2), 33% (a_3, b_3) in $CMBF_{L\text{-}Idi}$ (a) and $CMBF_{D\text{-}Alt}$ (b), and their stacking (a_4 and b_4).

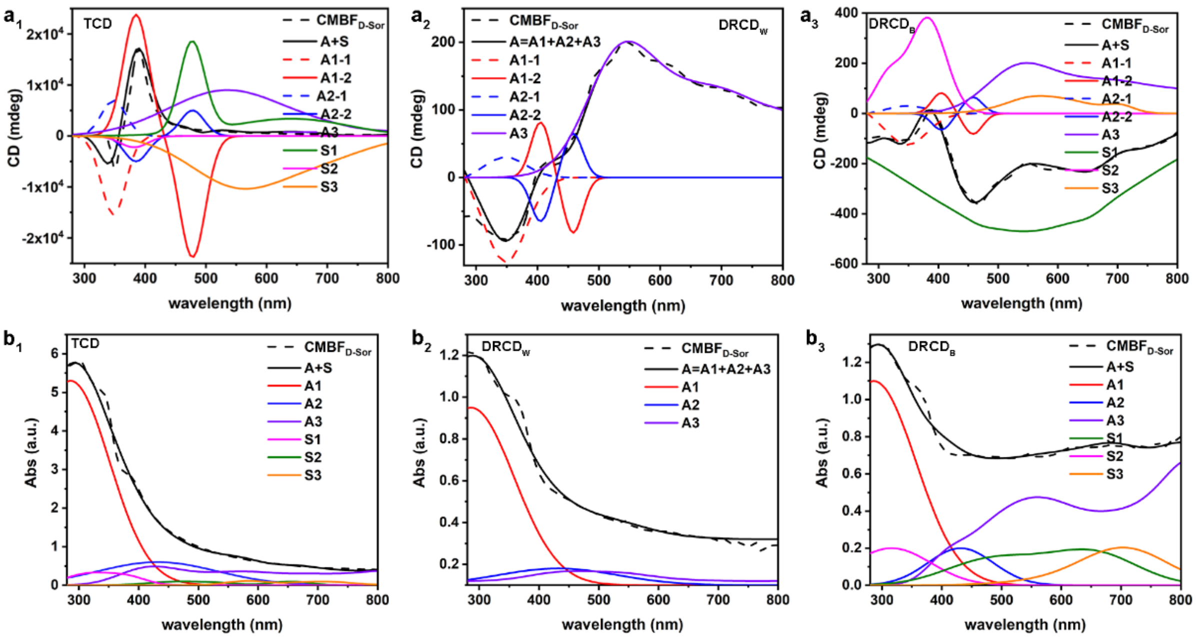

Figure S21. Peak differentiation-simulating analysis of TCD, DRCD with white ($DRCD_W$) and black ($DRCD_B$) (a), as well as the corresponding UV-Vis spectra (b) of $CMBF_{D\text{-}Sor}$. Schematic drawing summarizing the multiple OAs: (A1) AOA originates from the primary left-handed twisted nanoflakes. (A2) AOA originates from the secondary right-handed helical stacking of nanoflakes. (A3) AOA originates from the color response of the film. (S1) SOA originates from the primary left-handed twisted nanoflakes. (S2) SOA arises from secondary right-handed nanoflakes stacking. (S3) SOA arises from tertiary micrometer-sized vortexes with right-handed arrangement of nanoplates. The resulting CD signals and UV-Vis spectra are the superposed results of these OAs and extinction signals, respectively. The absorption of A1 and A2 shown in the UV-Vis spectra (Figure S21b) are actually the superimposed absorption of primary and secondary chirality.

Taking $CMBF_{D\text{-}Sor}$ as an example, three levels of chirality were confirmed: (i) primary left-handed nanoflakes with distorted crystal lattices at the atomic level (inset in Figure 3a_5), resulting in two negative CD signal derived Cotton effects ascribed to the electron transition absorption from O2p and Br4p to Bi6p (lines A1-1 and A1-2 in Figure S21) centered at approximately 286 and 431 nm and a positive CD signals corresponding to scattering across to 400-800 nm, respectively (line S1 in Figure S21); (ii) secondary nanoplates with right-handed helical stacking of nanoflakes (Figures 3a_4 and a_6), resulting in two positive electron transition-based CD signals ascribed to the electron transition absorption from O2p and Br4p to Bi6p (lines A2-1 and A2-2 in Figure S21) centered at approximately 286 and 431 nm and a negative SOA signal at 300-400 nm (line S2 in Figure S21); (iii) tertiary micron-sized vortexes composed of a right-handed arrangement of nanoplates (Figure 3a_3), resulting in a negative SOA signal at 400-800 nm (line S3 in Figure S21). We infer that the AOA signal at a wide visible range (line A3 in Figure S21) is due to the existence of a mid-gap state between the valence band top and conduction band tail.[13, 14] The UV-Vis and TCD spectra presented in Figure 4a are the superposed results of these extinction and OA signals, respectively.

Note：The fitting process is a simple extrapolation based on our experimental results. It assumes that the AOA and SOA are based on the theory of exciton couplet and circular Bragg resonance, respectively.

The exciton chirality rule occurs in chiral compounds containing at least two chromophores with an electric dipole allowed transitions.[15] If the chromophores are properly arranged with respect to each other, the molecule will display an exciton couplet CD signal (two CD bands with opposite sign and similar intensity), which arises from the through-space exciton coupling between the transition dipole moments (TDMs). Specifically, a positive couplet (a positive signal at lower energy and a negative signal at higher energy) indicates that the two TDMs define a right-handed arrangement, and vice versa. In our system, the nanounits can be regarded as chromophores, the chirality originates from the chiral arrangement of the these nanounits contribute to zero-crossover signal due to the exciton coupling. The left-handed

arrangement of nanounits selectively interact with circularly polarized (CP) light and produce a negative exciton coupling, while right-handed arrangement produce a positive exciton coupling.

The SOA originated from the circular Bragg resonance produces a CD band at the wavelength matching the pitch of the helical axis paralleled to incident light.[16] Commonly defined left-handed helices preferentially reflect left-handed CP light and transmit right-handed CP light, producing a negative DRCD signal and a positive TCD signal, and vice versa in right-handed helices.

According to electron crystallography and theoretical calculations, the chirality of each level is determined, thus, the shape of CD signals caused by each level of chirality is confirmed.

Because the primary and secondary chirality of CMBFs is smaller than the Bohr exciton radius, the excited state can be delocalized in the whole assembly, producing exciton coupling AOA signals. The tertiary chirality is too large to contribute to the AOA signal. However, three levels of chirality can exhibit SOAs at corresponding wavelengths that matched the pitches. Because of the different dispersion of pitches, the SOA signals might be broadened more or less.

Finally, the CD signals caused by each level of chirality are fitted according to the absorption spectrum of the CMBFs. Since both the UV/Vis and CD spectra are superimposed spectra with each acquisition point as the vertex of a Gaussian curve, we simulated each signal with a Gaussian function. The final fitting lines are the superposition of all Gaussian curves corresponding to those AOA and SOA arisen from each level of chirality.

A detailed simulation for using Origin 2018 is as follows: Graph → Add Functional Plot → Input Functions → Apply → Ok. According to the measured peak intensity of each CD signal to adjust the three parameters of a, b and c.

Table S1. Illustration of hierarchical chiralities and OAs in each level of chirality in $CMBF_{D\text{-}Sor}$.

Levels of structure	Handedness	AOA in CD spectrum	SOA in DRCD spectrum	SOA in TCD spectrum
Primary	Left-handedness	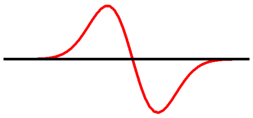 286 nm (O 2p→Bi 6p) 431 nm (Br 4p→Bi 6p)	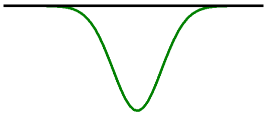 400-800 nm	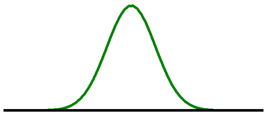 400-800 nm
Secondary	Right-handedness	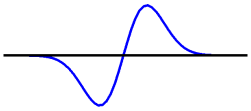 286 nm (O 2p→Bi 6p) 431 nm (Br 4p→Bi 6p)	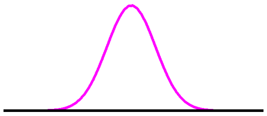 300-400 nm	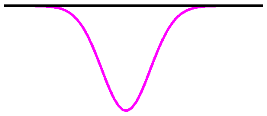 300-400 nm
Tertiary	Right-handedness	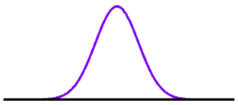 Visible range (colour response due to mid-gap state)	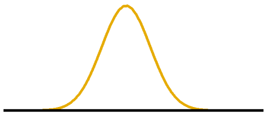 400-800 nm	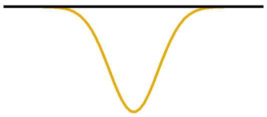 400-800 nm

Table S2. Descriptions of chiral basis and chirality of each level in CMBFs synthesized with different DSAs.

Samples	Asymmetric defects	Primary chirality	Secondary chirality	Tertiary chirality
$CMBF_{D\text{-}Sor}$	Four left-handed	Left-handed	Right-handed	Right-handed
$CMBF_{L\text{-}Idi}$	Two left- and one right-handed	Left-handed	Right-handed	Right-handed
$CMBF_{Gal}$	One left- and one right-handed	-	-	-
$CMBF_{All}$	-	-	-	-
$CMBF_{D\text{-}Man}$	Three right-handed	Right-handed	Left-handed	Left-handed
$CMBF_{D\text{-}Alt}$	Two right-handed	Right-handed	Left-handed	Left-handed

1. G. Kresse and J. Hafner, *Phys. Rev. B*, 1994, **49**, 14251.
2. J. P. Perdew, K. Burke and M. Ernzerhof, *Phys. Rev. Lett.*, 1996, **77**, 3865.
3. G. Kresse and D. Joubert, *Phys. Rev. B*, 1999, **59**, 1758.
4. S. L. Dudarev, G. A. Botton, S. Y. Savrasov, C. J. Humphreys and A. P. Sutton, *Phys. Rev. B*, 1998, **57**, 1505.
5. M. Assis, M. Carvalho de Oliveira, T. R. Machado, N. G. Macedo, J. P. C. Costa, L. Gracia, J. Andrés and E. Longo, *J. Phys. Chem. C*, 2019, **123**, 5023.
6. H. J. Monkhorst and J. D. Pack, *Phys. Rev. B*, 1976, **13**, 5188.
7. N. M. O'Boyle, M. Banck, C. A. James, C. Morley, T. Vandermeersch and G. R. Hutchison, *J. Cheminformatics*, 2011, **3**, 33.
8. A. K. Rappe, C. J. Casewit, K. S. Colwell, W. A. Goddard and W. M. Skiff, *J. Am. Chem. Soc.*, 1992, **114**, 10024.
9. A. C. Branka, *Phys. Rev. E: Stat. Phys., Plasmas, Fluids, Relat. Interdiscip. Top,* 2000, **61**, 4769.
10. W. Li, Y. Zou, X. Geng, F. Xiao, G. An and D. Wang, *Molecular Catalysis*, 2017, **438**, 19.
11. J.-M. Song, C.-J. Mao, H.-L. Niu, Y.-H. Shen and S.-Y. Zhang, *CrystEngComm*, 2010, **12**, 3875.
12. M. M. Ferrer, J. E. F. S. Rodrigues, M. A. P. Almeida, F. Moura, E. Longo, P. S. Pizani and J. R. Sambrano, *J. Raman. Spectr*, 2018, **49**, 1356.
13. X. Chen, L. Liu, P. Y. Yu and S. S. Mao, *Science*, 2011, **331**, 746.
14. A. Sinhamahapatra, J.-P. Jeon and J.-S. Yu, Energ. Environ. Sci., 2015, **8,** 3539.
15. G. Pescitelli, Chirality., 2021, 1–31.
16. M. Faryad, A. Lakhtakia, *Adv. Opt. Photonics,* 2014, **6,** 225.
